# Functions of Arginase Isoforms in Macrophage Inflammatory Responses: Impact on Cardiovascular Diseases and Metabolic Disorders

**DOI:** 10.3389/fimmu.2014.00533

**Published:** 2014-10-27

**Authors:** Zhihong Yang, Xiu-Fen Ming

**Affiliations:** ^1^Vascular Biology, Division of Physiology, Department of Medicine, Faculty of Science, University of Fribourg, Fribourg, Switzerland

**Keywords:** arginase, arginine, macrophages, nitric oxide synthase, cardiovascular diseases

## Abstract

Macrophages play a paramount role in immunity and inflammation-associated diseases, including infections, cardiovascular diseases, obesity-associated metabolic imbalances, and cancer. Compelling evidence from studies of recent years demonstrates that macrophages are heterogeneous and undergo heterogeneous phenotypic changes in response to microenvironmental stimuli. The M1 killer type response and the M2 repair type response are best known, and are two extreme examples. Among other markers, inducible nitric oxide synthase and type-I arginase (Arg-I), the enzymes that are involved in l-arginine/nitric oxide (NO) metabolism, are associated with the M1 and M2 phenotype, respectively, and therefore widely used as the markers for characterization of the two macrophage phenotypes. There is also a type-II arginase (Arg-II), which is expressed in macrophages and prevalently viewed as having the same function as Arg-I in the cells. In contrast to Arg-I, little information on the role of Arg-II in macrophage inflammatory responses is available. Emerging evidence, however, suggests that differential roles of Arg-I and Arg-II in regulating macrophage functions. In this article, we will review recent developments on the functional roles of the two arginase isoforms in regulation of macrophage inflammatory responses by focusing on their impact on the pathogenesis of cardiovascular diseases and metabolic disorders.

## Introduction

Macrophages are important sentinel cells in our body and are involved in maintenance of tissue homeostasis, immune responses, and inflammation-associated diseases. Recent findings have revised our traditional view on the origin and biological functions of macrophages. We now know that tissue macrophages are not only recruited from bone marrow-derived monocytes but also differentiated from yolk sac-derived embryonic stem cells (1–3). Moreover, tissue macrophages are not terminally differentiated and are maintained throughout life by local proliferative self-renewal (4, 5). Importantly, macrophages are highly heterogeneous and undergo phenotypic changes, i.e., macrophage plasticity, in response to specific signals as a consequence of adaptation to local tissue environmental cues (6, 7). The original and the best known types of macrophage responses are the pro-inflammatory M1 type (killer cells) and the anti-inflammatory M2 type (repair type cells) (3, 7). There are convincing evidences from research of recent years showing that different phenotypic macrophages are indeed importantly participating in the process of immune and inflammatory responses, which have been reviewed by many comprehensive articles (7, 8).

## Macrophage Polarization

Macrophage polarization describes acquirement of distinctive phenotypic and functional characteristics of fully differentiated macrophages in response to microenvironmental stimuli. Functional polarization of macrophages and the underlying mechanisms that control the cell phenotypes are complex and have been extensively investigated in recent years. As mentioned, the M1 and M2 classifications of macrophages described the two major and opposing activities committed to killing and repairing functions of the cells. It is emerging that macrophage polarization is regulated by a broad spectrum of recognition receptors, cytokines, specific signaling pathways, and genetic programs. Some of them are used as markers or functional repertoire of the macrophage phenotypes. There are, however, no standard guidelines for classification of macrophage phenotypes. Most importantly, information about functions of these markers in regulation of macrophage inflammatory responses or phenotypes is either lacking or controversial. The conclusions are usually based on association studies. It is generally the view that M1 macrophages express enhanced genes, which are pro-inflammatory and cytotoxic, typically inducible nitric oxide synthase (iNOS)/NO, IL-12, class II MHC, and the chemokines IL-8 and CCL2, participating in killing intracellular parasites and tumor development. In contrast, M2 macrophages produce more anti-inflammatory cytokines and substances involved in repairing function, typically, arginase/ornithine, EGF, VEGF, and TGF-β, and mannose receptor (9). This phenotypic cell is mainly participating in resolution of inflammation, tissue repairing, angiogenesis, allergy, and tumor progression (10). It is, however, to notice that M1 and M2 activation programs display differences, but they may not form clear-cut activation subsets and reveal overlapping effects. A discussion about the complexity of macrophage phenotype markers, differentiation mechanisms, and the roles in human diseases is beyond the scope of this review article. For these aspects, readers are kindly asked to refer to several comprehensive review articles (11, 12). In the following section of this article, we will focus on discussing the role of the enzymes arginase and nitric oxide synthase (NOS) that are involved in l-arginine metabolism in various cell types including vascular endothelial cells and macrophages and widely used as markers to distinguish M1 and M2 macrophage phenotypes.

## l-Arginine Metabolism, iNOS, and Arginase in Macrophage Functional Polarization

**Figure 1:**
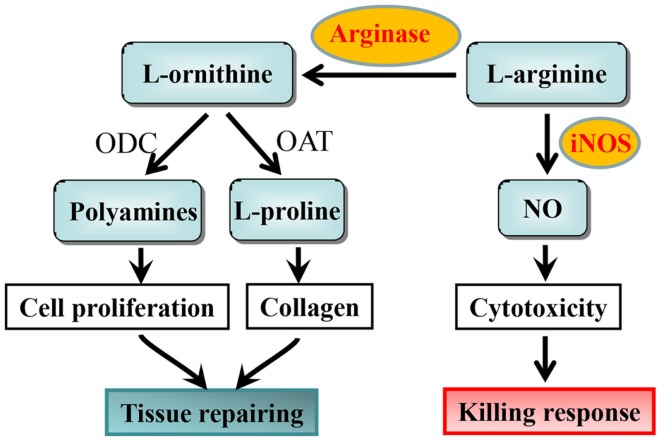
**l-arginine metabolism by iNOS and arginase and the functional consequences in macrophages**. ODC, ornithine decarboxylase; OAT, ornithine aminotransferase.

The suggestion that l-arginine metabolism could be involved in regulation of macrophage phenotypes was from early studies with macrophages isolated from the Th1 strain mouse C57B1/6 and Th2 strain BALB/c mouse (13, 14). These studies demonstrate that isolated macrophages from Th1 strain C57Bl/6 mouse are more readily activated to produce nitric oxide (NO) upon stimulation with IFN-γ or lipopolysaccharide (LPS) than the macrophages from Th2 strain BALB/c mouse. Later on, it was characterized that M1 macrophages are more easily activated by LPS to produce cytotoxic NO via iNOS, whereas M2 macrophages generate little NO but more ornithine from the same substrate l-arginine via arginase (15). The iNOS and arginase are thought to affect inflammatory responses in the opposite way. NO production from iNOS in M1 macrophages inhibits cell proliferation and kills pathogens, a M1 killing type response (16, 17), while ornithine production promotes cell proliferation and repairs tissue damage through generation of polyamines and collagen in M2 macrophages, a M2 repairing type response (11, 18). Both NO and ornithine are generated from the same substrate l-arginine via iNOS and arginase, respectively (11, 18) (Figure 1). From these studies, one can consider dominant NO production as M1 activity, whereas dominant ornithine production as M2 activity of macrophages.

## Arginase Isoenzymes and l-Arginine Metabolizing Functions

In human beings and mammals, there are two isoforms of arginases, arginase-1 (Arg-I) and arginase-II (Arg-II). Both isoenzymes are encoded by two separate genes. In human beings, Arg-I gene maps to chromosome 6q23 and encodes a 322 amino acid protein (19–21), while Arg-II gene maps to chromosome 14q24.1 and encodes a 354 amino acid protein (22–24). At the subcellular level, Arg-I is mainly localized in cytoplasm and Arg-II in mitochondrion (25). The physiological role of the different subcellular compartmentation of the two isoenzymes is not known. The two isoenzymes, however, share similar structure, reveal more than 50% of homology of their amino acid residues with 100% homology in the areas, which are critical for their l-arginine metabolizing function (22, 23, 26). Although both Arg-I and Arg-II are to hydrolyze l-arginine to produce urea and l-ornithine (25), the functional impact of the two isoenzymes is either similar or different depending on specific organs/cells. For example, increased activity and/or expression of either Arg-I or Arg-II in endothelial cells impair the vasoprotective endothelial NO production via eNOS (27). However, in macrophages, Arg-I and Arg-II seem to play an opposite function, which we will discuss later in this article. The primary function of Arg-I is to remove excessive nitrogen produced from amino acid metabolism through hepatic urea cycle, which is otherwise toxic for our body (28, 29), because Arg-I is constitutively and abundantly expressed as a cytosolic enzyme in the liver (30). No Arg-II could be detected in hepatocytes. The vital effect of hepatic Arg-I is evidenced by the studies showing that Arg-I knockout mice reveal severe symptoms of hyperammonemia and die between postnatal days 10 and 14 (31). Patients with Arg-I deficiency due to gene mutation reveal urea cycle disorder, hyperargininemia, and exhibit progressive neurologic impairment, development retardation, and hepatic dysfunction associated with cirrhosis and carcinoma in early childhood (28, 29). Arg-I has been reported to be expressed also in many extrahepatic tissues such as stomach, pancreas, and lung (32). The functions of Arg-I in these organs are far from clear. Unlike Arg-I, Arg-II is confined mainly to kidney, brain, prostate, intestine, and also pancreas (22, 23, 32). The functions of Arg-II in these organs are not known. The best characterization of Arg-II function is done in vascular endothelial cells in which the isoenzyme, similar to Arg-I, metabolizes l-arginine to urea and l-ornithine, which limits l-arginine bioavailability for generation of the vasoprotective NO via eNOS, resulting in vascular endothelial dysfunction (33, 34). This effect of arginases on endothelial cells is attributable to eNOS-uncoupling, a situation that eNOS enzyme produces increased superoxide anion, but decreased NO (34–39), which is thought to be attributed to l-arginine deficiency, leading to oxidative stress, and enhanced expression of endothelial inflammatory adhesion molecules such as VCAM-1 and ICAM-1 (39), since endothelial NO reveals important anti-oxidative and anti-inflammatory functions and suppresses expression of the adhesion molecules (40). These effects of Arg-II are dependent on the enzymatic activity, since loss-of-function point mutation of histidine to phenylalanine at position 160 in Arg-II abolishes its l-arginine–urea hydrolase activity and is unable to cause eNOS-uncoupling and the inflammatory responses in endothelial cells (39). We have recently reviewed the aspect of arginase in eNOS-uncoupling (41). Arginase also exerts pleiotropic effects, i.e., l-arginine–urea hydrolase activity-independent effect, which we will discuss later in this article.

## Arginase Isoenzymes and Macrophage Functions

As mentioned above, macrophages are heterogeneous and undergo phenotypic changes, depending on microenvironmental stimuli. The expression of Arg-I and Arg-II is inducible in macrophages depending on external stimuli (42, 43). As discussed, NO from iNOS in macrophages is linked to M1, whereas ornithine generated from arginase is associated with M2 phenotype (11). Substantial number of studies demonstrates that Arg-I is dominantly expressed in M2 cells and reduces NO production from iNOS through limiting bioavailability of intracellular l-arginine, resulting in dampening of inflammatory tissue damage and suppression of clearance of intracellular pathogens (44–48). In contrast to Arg-I, only very little and even contradictory information is available about the expression and role of Arg-II in macrophage phenotype regulation and inflammatory responses. Until we have systematically investigated this specific aspect in macrophage inflammatory responses (43), the function of Arg-II in macrophages is believed to be anti-inflammatory, which is extrapolated from its similar function as Arg-I on l-arginine/NO metabolism. An early study showed that Arg-II gene is a direct target of liver X receptor that has been shown to exert inhibitory effects on expression of inflammatory genes in macrophages (49). Based on this association, the authors suggest that Arg-II is anti-inflammatory. The functional analysis is, however, not done. It is of particular importance to note that LPS stimulation exclusively enhances iNOS in macrophages associated with M1 phenotype (43, 50). We could demonstrate that iNOS induction in macrophages is paralleled with enhanced expression of Arg-II, but not Arg-I (43), which suggests that Arg-I and Arg-II shall have different functions in macrophage inflammatory responses or phenotype regulation. In line with this observation, accumulation of Arg-II-expressing macrophages is associated with advanced atherosclerotic lesions in which pro-inflammatory cells are dominant (42), suggesting that Arg-II is associated with pro-inflammatory responses. Because of this contradictory concept about the role of Arg-II and lack of functional analysis of Arg-II in macrophage inflammatory responses, we recently systematically characterized the role of Arg-II in regulation of macrophage inflammations at the cellular and whole body levels in mouse models of chronic inflammatory diseases such as obesity-linked insulin resistance, type-II diabetes mellitus, and atherosclerosis (43).

**Figure 2:**
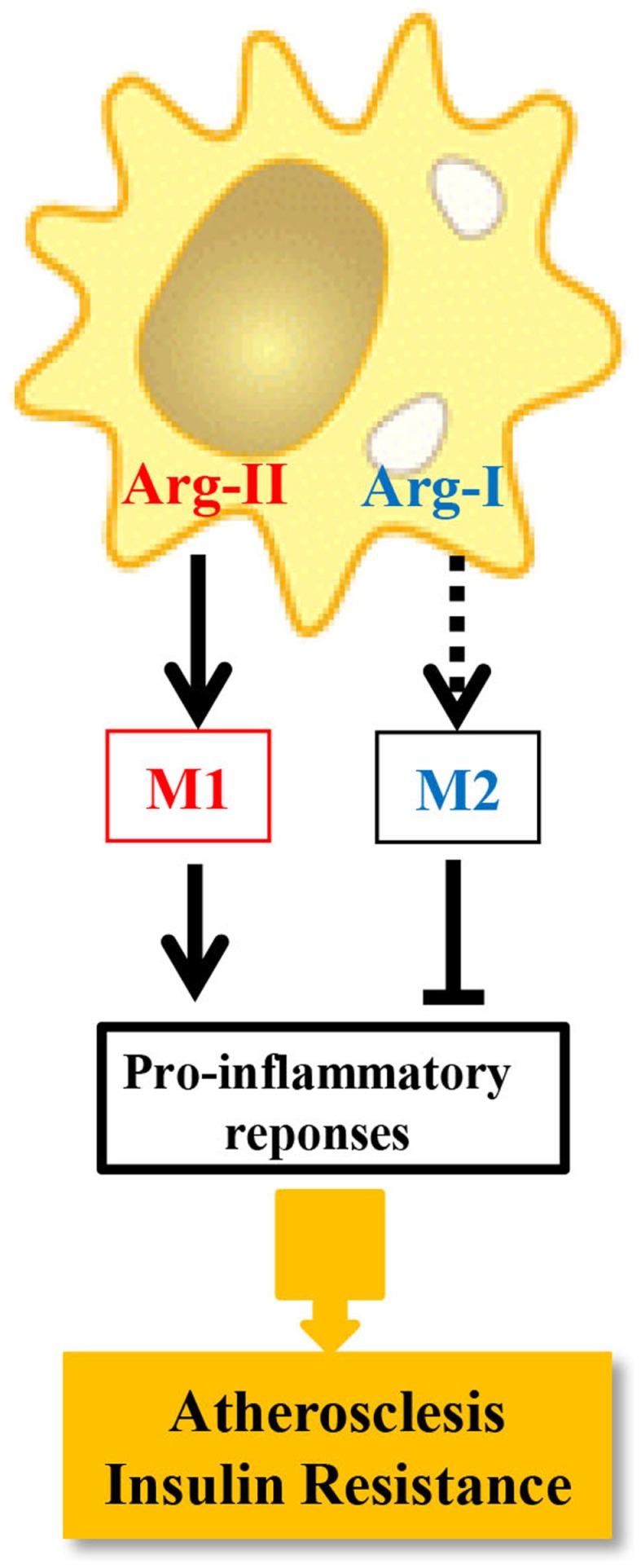
**The distinct role of Arg-I and Arg-II in macrophage inflammatory responses**. Dashed line indicates that the causal role of Arg-I in M2 phenotype requires determination.

In this study, we demonstrate that M1 activation of macrophages by LPS exclusively up-regulates iNOS and Arg-II, but not Arg-I expression in murine and human macrophages (43). Silencing Arg-II gene in human monocyte/macrophage cell lines decreases the cell adhesion to endothelial cells with reduced production of pro-inflammatory cytokines in response to LPS or ox-LDL at both the mRNA and protein levels. Moreover, LPS-induced up-regulation of numerous pro-inflammatory mediators, including MCP-1, TNF-α, IL-6, MMP14, and iNOS, is significantly suppressed in macrophages isolated from Arg-II^−/−^ mice as compared with those from wild-type control animals. Convincingly, introducing Arg-II gene back to the Arg-II^−/−^ macrophages restores or enhances the LPS-stimulated expression of the pro-inflammatory genes to much higher levels compared to the Arg-II^+/+^ cells from wild-type mice. Importantly, Arg-II^−/−^ mice are protected from systemic pro-inflammatory macrophage infiltration in various organs and expression of pro-inflammatory mediators in high-fat diet (HFD)-induced obesity. Arg-II^−/−^ mice, when fed a HFD, although have similar body weight as WT controls, reveal lower fasting plasma glucose concentration, are more glucose tolerant and insulin sensitive (43) as compared to WT mice on HFD. Interestingly, Arg-II levels in macrophages are significantly increased in WT mice fed HFD, which is associated with pro-inflammatory responses. The pro-inflammatory function of Arg-II in macrophages is further demonstrated in another chronic inflammatory disease model, i.e., atherosclerosis mouse model (43). Knocking-out Arg-II gene in the atherosclerosis-prone ApoE^−/−^ mice (ApoE^−/−^/Arg-II^−/−^) decreases inflammatory cytokine levels and macrophage content in the aortas, reduces atherosclerotic plaque formation, and reveals more stable plaque features as compared to ApoE^−/−^Arg-II^+/+^ control mice. Since M1 pro-inflammatory macrophages play crucial role in development of insulin resistance and type-II diabetes and atherogenesis (51–55), our results demonstrate that Arg-II promotes pro-inflammatory or M1 phenotype of macrophages and favors chronic inflammatory disease development such as obesity-associated insulin resistance, type-II diabetes, and atherosclerosis. It is to mention that the pro-inflammatory effect of Arg-II in macrophages does not seem to be relying on iNOS, since inhibition of iNOS does not significantly affect expression of several pro-inflammatory genes in macrophages. The dissociation of arginase activity from iNOS has been reported by several studies, showing that alteration of arginase activity in macrophages is not necessarily associated with functional changes in iNOS (56–59). These iNOS-independent pro-inflammatory responses mediated by Arg-II in macrophages are due to enhanced mitochondrial ROS, since reintroduction of the Arg-II gene into Arg-II^−/−^ macrophages enhances mitochondrial $O_{2}^{•-}$ and H_2_O_2_ generation and inhibition of mitochondrial ROS significantly reduces Arg-II-mediated inflammatory responses. The function of Arg-II in comparison with Arg-I in macrophage inflammatory responses and chronic inflammatory diseases, i.e., atherosclerosis and insulin resistance is summarized in Figure 2. It is not very surprising, since Arg-II is a mitochondrial enzyme (60). The question remains elusive how Arg-II affects mitochondrial function leading to mitochondrial ROS production in macrophages.

Although we have characterized the function of Arg-II in macrophages, many important questions remain unanswered. In the following section, we will briefly discuss several important remaining questions regarding the role of arginase isoenzymes in macrophage functions.

## Future Research Questions and Perspectives

### Does Arg-I play a causal role in M2 macrophage phenotype?

As discussed, Arg-I is constitutively expressed in hepatocytes and is inducible in macrophages, e.g., by Th2 cytokines IL-4 and IL-13 (44, 61, 62). It is highly upregulated in M2 macrophages and widely used as a M2 macrophage marker (11). It has been suggested that Arg-I in macrophages promotes Th2 cytokine production, contributing to resolution of inflammation and tissue repairing (63). A study in human smooth muscle cells showing that overexpression of Arg-I gene is able to decrease LPS-induced pro-inflammatory cytokine production (64), seems to support the anti-inflammatory function of Arg-I. However, mice with specific Arg-I gene deficiency in macrophages show exacerbated Th2 response and fibrosis in the liver of *Schistosoma mansoni* infected mice (65), which does not seem to support previous suggestions in promotion of Th2 responses (63). Most studies demonstrate only the positive correlation of Arg-I with M2 macrophage phenotype, the causal relationship of Arg-I with macrophage phenotype is, however, not fully clear. Importantly, a recent study demonstrates that Arg-I deficient mouse macrophages has even higher polyamine production and does not impair gene expression in response to IL-4 (66), which raises the question about the role of Arg-I in M2 macrophage regulation. Future research shall elucidate the causal role of Arg-I in regulation of macrophage functional polarization.

### Does l-arginine deficiency explain the functions of arginase isoenzymes in macrophages?

There is continuing debate about the role of l-arginine deficiency in arginase-induced alterations of cellular functions. It is generally believed that arginase including Arg-I and Arg-II causes l-arginine deficiency, resulting in decreased NO production from eNOS in endothelial cells (endothelial dysfunction) and from iNOS in macrophages (M2 type function) (41). It has been demonstrated that the concentration of l-arginine in adult human and mouse plasma (0.1 mmol/L), as well as intracellular arginine concentration (0.05–0.2 mmol/L) far exceed the *K_m_* of eNOS (2–20 μmol/L) (67). Even though, acute l-arginine supplementation in cells, isolated blood vessels, or in animals or in patients is able to enhance NO production and improve endothelium-dependent relaxations, a situation called “arginine paradox” (68, 69). This phenomenon led to doubt whether l-arginine deficiency caused by arginase is true. Several hypotheses have been proposed to explain the “arginine paradox.” First, a “relative” intracellular l-arginine deficiency, resulting from an increased level of endogenous competitors for eNOS substrate l-arginine such as ADMA that binds to eNOS but could not be metabolized by the enzyme on top of increased arginase activity either Arg-I or Arg-II in endothelial cells has been suggested (70). Experiments showed that inhibition of arginase improves eNOS function and overexpression of Arg-I or Arg-II causes eNOS-uncoupling, leading to oxidative stress, and decreased bioavailability of endothelial NO production, which is associated with only 11–25% reduction in intracellular l-arginine concentration in the presence of high-extracellular concentration of l-arginine (0.4 mmol/L) (71). These results seem to support the “relative l-arginine deficiency” hypothesis. It is worthy of noting that NO production, particularly, iNOS/NO can be inhibited by TGF-β, which is a strong Arg-I up-regulator and present in very high amount during wound healing (72). It is presumable that NO production is inhibited even under the condition of high-plasma l-arginine concentration because of high concentration of TGF-β. Whether this could explain the “arginine paradox” is not known. Another alternative hypothesis is that a specific intracellular pool of l-arginine for NO production may exist in endothelial cells and could be depleted by enhanced arginase (73), yet, it is highly speculative. It is not known whether enhanced arginase activity, particularly Arg-II, could also cause iNOS-uncoupling, affecting macrophage functions. Another puzzling is that why Arg-I and Arg-II share the same l-arginine metabolizing function but seem to exert distinct effects on macrophages.

If there is no real l-arginine deficiency caused by arginase either Arg-I or Arg-II, l-arginine supplementation therapy aiming to enhance endothelial NO production and to treat vascular disease shall not work. In accordance, clinical studies in patients with acute myocardial infarction or with peripheral arterial disease demonstrate that 6-month oral l-arginine supplementation (3 g three times a day on top of standard medications) increase mortality and shorten walking distance accompanied with decreased NO production as compared to the placebo group (74). The underlying mechanisms are not known and may be related to the induction of arginase, particularly Arg-II in vascular endothelial cells by chronic l-arginine exposure as demonstrated by recent studies including our own (37, 69). These studies show that acute supplementation of l-arginine to endothelial cells increases NO bioavailability, while chronic l-arginine supplementation causes eNOS-uncoupling mediated by up-regulation of Arg-II levels, leading to endothelial senescence (69). Similar to this finding, l-arginine has also been reported to cause iNOS-uncoupling in macrophages (75). These studies do not support a role of absolute l-arginine deficiency caused by arginase, but strongly implicate that too much l-arginine is even harmful, which is probably due to production of other undesired metabolites from l-arginine as speculated (76). Alternatively, a pleiotropic effect may also provide explanation for the detrimental effects of arginase at least for Arg-II under the condition of sufficient l-arginine supply. This point will be discussed below.

### Does arginase exert pleiotropic effects: l-arginine–urea hydrolase activity-independent effects?

**Figure 3:**
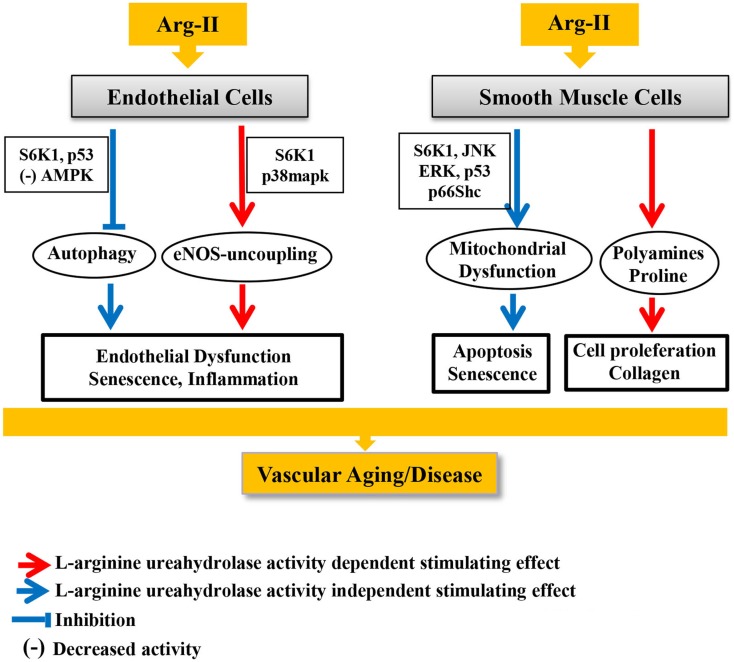
**Canonical and pleiotropic effects of Arg-II in vascular endothelial and smooth muscle cells and underlying signaling mechanisms in development of vascular aging and age-associated vascular diseases**.

Any proteins or enzymes may have pleiotropic or off-target effects that are not necessarily related to their canonical functions. We have recently discovered that Arg-II exhibits its biological functions in vascular cells through both mechanisms, which are either dependent or independent on l-arginine metabolizing function (l-arginine–urea hydrolase activity) (77). We show that the catalytically inactive mouse Arg-II mutant with a point mutation of histidine to phenylalanine at position 160 (referred to as H160F), which lost its l-arginine–urea hydrolase activity, although does not cause eNOS-dysfunction in endothelial cells, promotes cell apoptosis and senescence to the same extent as the WT Arg-II in vascular smooth muscle cells (VSMC). In contrast, only the WT Arg-II (not the H160F inactive mutant) exerts function to promote VSMC proliferation (Figure 3), which can be attributed to the production of polyamine from l-arginine/ornithine pathway. This intriguing result provides evidence that Arg-II on one hand promotes VSMC proliferation and on the other hand causes VSMC apoptosis and senescence. While the cell proliferation–stimulating effect of Arg-II is dependent on its l-arginine–urea hydrolase activity via synthesis of ornithine and polyamines (71, 78), the cell apoptosis/senescence-promoting effect is independent of its enzymatic activity (Figure 3). Further experiments show that this l-arginine–urea hydrolase activity-independent effect is mediated through signaling pathways including mTORC1/S6K1, JNK, and ERK1/2, converging on p66^Shc^ leading to H_2_O_2_ production and mitochondrial dysfunction leading to cellular apoptosis and senescence (77) (Figure 3). In parallel to these signaling pathways, p53 is also activated by Arg-II independently of its l-arginine–urea hydrolase activity, contributing to the cell senescence of the apoptosis process. Importantly, expression of Arg-II and activities of S6K1, ERK1/2, p66Shc, and p53 are all augmented in senescent VSMC, and genetic inhibition or ablation of Arg-II not only reduces these signaling pathways and VSMC senescence/apoptosis *in vitro* but also in atherosclerosis-prone ApoE^−/−^ mice *in vivo*, which at least in part accounts for the reduced plaque lesion formation and a more stable plaque characteristics in aortic roots in Arg-II-deficient ApoE^−/−^ mice (43, 77) (Figure 3). Moreover, we also show that Arg-II negatively regulates autophagy function – a cell protective mechanism of lysosomal proteolysis aiming to remove harmful proteins (79) – in endothelial cells, which is also independent on its l-arginine–urea hydrolase activity [Ref. (69), Figure 3]. Decreased autophagy function in vascular cells and macrophages are implicated in vascular aging and atherosclerotic vascular disease. Indeed, recent studies provide evidence suggesting that adequate induction of autophagy protects against cellular injury in endothelial and smooth muscle cells and formation of foam cells, resulting in anti-atherosclerotic effects (80–82). In line with this finding, genetic ablation of Arg-II in atherosclerotic ApoE^−/−^ mice preserves endothelial autophagy in aortas, which associates with reduced atherosclerosis lesion formation (69). In this study, we also demonstrate that Arg-II impairs endothelial autophagy independently of the l-arginine–urea hydrolase activity through activation of mTORC1/S6K1 and p53, resulting in inhibition of AMPK in endothelial cells, which contributes to development of atherosclerosis (Figure 3). How Arg-II, independently of its l-arginine–urea hydrolase activity, impacts vascular cell functions, remains to be investigated. Further, whether these enzymatic dependent and independent effects also exist for Arg-I and whether the pleiotropic effects of arginase account for functional regulations in macrophages are unknown.

### What are the mechanisms that regulate Arg-I and Arg-II in macrophages

Arginase-I gene expression is inducible in macrophages by a variety of stimuli, for example, by elevated cAMP, IL-4, and TGF-β (50). The regulation of Arg-I gene expression is mainly investigated at the transcriptional levels in murine macrophages, while it remains to be investigated whether the findings are also true in human cells. A number of transcription factors and nuclear receptors such as RXR, PPARγ, PPAR δ, STAT6, C/EBPβ, KLF4, PU.1, IRF8, and AP-1 have been shown to bind directly to specific sites in the promotor region of Arg-1 gene and in turn to enhance Arg-1 expression. The complexity of Arg-I gene regulation mechanisms are further complicated by the fact that these transcription factors can be regulated by post-translational modification mechanisms such as SUMOylation and ubiquitination that are participating in the regulation of Arg-1 gene [for detailed description of these mechanisms please refer to the review articles (83, 84)]. There is, however, little information available regarding the upstream regulatory mechanisms involved in gene expression and enzymatic activity of Arg-I in macrophages. Also, very limited information is provided on regulation of Arg-II in macrophages. The stress sensor p38mapk has been demonstrated to participate in up-regulation of activity and expression of Arg-I and Arg-II in macrophages (85, 86). This seems to be also the case in bovine and rat aortic endothelial cells for Arg-I expression (87) and in human endothelial cells and mouse penile tissues for Arg-II (88, 89). In accordance, *in vivo* treatment of hypertensive mouse induced by angiotensin-II infusion with a p38mapk inhibitor prevents elevation of Arg-II expression and activity and enhances endothelium-dependent relaxation (88). These studies demonstrate that p38mapk is the upstream regulator of Arg-II in endothelial cells and macrophages. Our most recent study provides evidence showing that p38mapk also functions as downstream effector of Arg-II in endothelial cells, causing oxidative stress through eNOS-uncoupling, since overexpression of Arg-II in human endothelial cells causes eNOS-uncoupling and augments p38mapk activation (90), and inhibition of p38mapk either pharmacologically by SB203580 or genetically by silencing the major isoform p38mapkα in endothelial cells prevents eNOS-uncoupling effect by Arg-II gene overexpression (90). Furthermore, mice fed HFD, an obesity mouse model, exhibit enhanced Arg-II expression/activity and p38mapk activity and eNOS-uncoupling in the aortas and inhibition of p38mapk recouples eNOS activity in the obese mice. Moreover, mice deficient in Arg-II (Arg-II^−/−^) on the same obesogenic diet reveal decreased p38mapk activity and eNOS function is fully preserved. These results demonstrate that Arg-II causes eNOS-uncoupling through activation of p38mapk in HFD-induced obesity (90). Together with the experiments discussed above, there might be a positive regulatory circuit between p38mapk and Arg-II at least in vascular endothelial cells. Whether this mechanism is also involved in Arg-I and/or Arg-II gene expression in macrophages is not known. A similar positive regulatory circuit between S6K1 and Arg-II has also been demonstrated by our recent studies in vascular endothelial cells (39).

In this study, we show that a persistent hyperactive S6K1 activity is found to play a causal role in eNOS-uncoupling, leading to vascular endothelial aging and senescence (39, 91). Overexpression of a constitutively active S6K1 mutant up-regulates Arg-II (not Arg-I) gene expression and arginase activity in non-senescent cells by stabilizing Arg-II mRNA (39). Conversely, silencing S6K1 in senescent cells reduces Arg-II gene expression and activity and genetic or pharmacological inhibition of S6K1 in senescent cells or in old rat aortas decreases Arg-II gene expression and activity, demonstrating a critical role of hyperactive S6K1 in up-regulating Arg-II gene expression, resulting in enhanced arginase activity in endothelial aging. Interestingly, silencing Arg-II gene in senescent endothelial cells or deficiency in Arg-II gene in mice reduces S6K1 activity, recouples eNOS function in aging, and inhibits endothelial expression of adhesion molecules such as ICAM-1 and VCAM-1, resulting in inhibition of monocyte-endothelial cell interaction, demonstrating a positive vicious cycle between S6K1 and Arg-II in vascular endothelial aging. These studies provide evidence showing that a mutual positive regulation between S6K1 and Arg-II gene expression accelerates endothelial aging through eNOS-uncoupling, leading to oxidative stress and inflammation (39). Further studies will analyze whether S6K1 is also involved in regulation of Arg-I and/or Arg-II in macrophages, participating in macrophages phenotype determination. Also, the relationship between p38mapk, S6K1, and arginase remain to be analyzed.

Other signaling pathways such as GTPase RhoA and its downstream kinase ROCK have been reported to mediate Arg-I gene expression in porcine coronary arterioles in response to hydrogen peroxide (H_2_O_2_) and peroxynitrite (92) and Arg-II (but not Arg-I) expression and/or activity in women with preeclampsia (93) and in human endothelial cells in response to thrombin (35), oxidized LDL (94), and hyperglycemia (36). In macrophages, however, ROCK kinase inhibitor enhances Arg-I expression and shift M1 to M2 phenotype (95), suggesting that ROCK pathway may inhibit Arg-I expression. No information is available so far whether Rho/ROCK pathway is involved in Arg-II regulation in macrophages. For the detailed regulatory signaling mechanisms of Arg-I and Arg-II expression/activity in vascular cells, please refer to the review article (41).

## Conclusion

The two isoforms of arginase, i.e., Arg-I and Arg-II, although located in different subcellular compartments, share the same function on l-arginine metabolism. Both isoenzymes hydrolyze l-arginine to urea and l-ornithine, resulting in eNOS-uncoupling in endothelial cells. In macrophages, Arg-I and Arg-II can be differentially induced by external stimuli. Evidence has been provided that Arg-II plays a causal role in M1 functions, whereas Arg-I is associated with M2 function in macrophages and widely used as M2 marker for macrophages. However, the causal relationship between Arg-I and M2 phenotype warrants further investigation. It remains to be characterized how Arg-I and Arg-II share the same l-arginine metabolizing effect, but exhibit distinct or opposite effects in macrophage inflammatory responses. Arg-II as therapeutic target in chronic inflammatory disorders such as age-associated vascular dysfunctions, atherosclerosis, and type-II diabetes and complications has shown promising beneficial effects in genetic modified mouse models (39, 43, 96). Some studies implicate that targeting Arg-I is also beneficial for cardiovascular functions, these studies are solely dependent on the pharmacological inhibitors, which inhibit both isoforms of arginases (97–99), since systemic Arg-I deficient mouse exhibits severe symptoms of hyperammonemia, and die between postnatal days 10 and 14 (31), one should consider that these inhibitors could inhibit liver Arg-I, resulting in hyperammonemia. Taking into account that Arg-I in macrophages may exhibit opposite effects as Arg-II, this is another important reason to develop specific Arg-II inhibitors. Moreover, whether Arg-I and Arg-II exert pleiotropic effects on macrophage functions as demonstrated in vascular cells shall be investigated. If this proves to be true, development of therapeutic drugs that target l-arginine–urea hydrolase activity may have limitation on treatment of inflammatory diseases. Additionally, signaling pathways that are involved in regulation of gene expression and enzymatic activity of both Arg-I and Arg-II shall be further elucidated in macrophages. Characterization of these signaling mechanisms will also provide possibilities or rationales to target arginase isoforms specifically in an indirect way to treat inflammatory diseases. Finally, we have focused on cardiovascular and metabolic diseases here. But, functional analysis of arginase isoenzymes and their roles in macrophage polarization should also help understanding other diseases, notably cancer. In particular, monocytes and macrophages are recruited into tumors and regulate tumor growth by changing their functional phenotypes, which is originally demonstrated by Mills and colleagues (100). M1 macrophage has been shown to have antitumor immunity, whereas the M2 macrophage exerts protumorigenic properties (101). Regardless of the inflammatory circumstance, it appears that macrophage arginases are key players in influencing disease outcomes.

## Conflict of Interest Statement

The authors declare that the research was conducted in the absence of any commercial or financial relationships that could be construed as a potential conflict of interest.
